# Comparison of the cytokine adsorption ability in continuous renal replacement therapy using polyethyleneimine-coated polyacrylonitrile (AN69ST) or polymethylmethacrylate (PMMA) hemofilters: a pilot single-center open-label randomized control trial

**DOI:** 10.1186/s40001-023-01184-6

**Published:** 2023-06-30

**Authors:** Yoshihiko Nakamura, Hiroki Hatomoto, Shintaro Yamasaki, Kazuya Yamauchi, Fumiaki Kiyomi, Kota Hoshino, Yasumasa Kawano, Takafumi Nakano, Takehiro Hasegawa, Hiroyasu Ishikura

**Affiliations:** 1 Department of Emergency and Critical Care Medicine, Fukuoka University Hospital, 7-45-1 Nanakuma, Jonan-ku, Fukuoka, 814-0180 Japan; 2 Department of Clinical Engineer Center, Fukuoka University Hospital, Fukuoka, Japan; 3 Clinical Research Support Center Kyusyu, Fukuoka, Japan; 4 Department of Pharmacology, Faculty of Pharmaceutical Sciences, Fukuoka University, Fukuoka, Japan; 5 Research and Development Division, Sysmex R&D Centre Europe GmbH, Hamburg, Germany

**Keywords:** Sepsis, Cytokine adsorption, Continuous renal replacement therapy, Polyethyleneimine-coated, Polymethylmethacrylate

## Abstract

**Background:**

Sepsis occurs as a result of dysregulated host response to infection. However, cytokine adsorption therapy may restore the balance of proinflammatory and anti-inflammatory mediator responses in patients with sepsis. This study aimed to determine the cytokine adsorption ability of two different types of continuous renal replacement therapy (CRRT) hemofilters for polyethyleneimine-coated polyacrylonitrile (AN69ST) (surface-treated) and polymethylmethacrylate (PMMA) CRRT.

**Methods:**

We performed a randomized controlled trial among sepsis patients undergoing CRRT, who were randomly assigned (1:1) to receive either AN69ST or PMMA-CRRT. The primary outcome was cytokine clearance of hemofilter adsorption (CHA). The secondary endpoints were the intensive care unit (ICU) and 28-day mortalities.

**Results:**

We randomly selected 52 patients. Primary outcome data were available for 26 patients each in the AN69ST-CRRT and PMMA-CRRT arms. The CHA of high-mobility group box 1, tumor necrosis factor, interleukin (IL)-8, monokine induced by interferon-γ, and macrophage inflammatory protein were significantly higher in the AN69ST-CRRT group than in the PMMA-CRRT group (*P* < 0.001, *P* < 0.01, *P* < 0.001, *P* < 0.001 and *P* < 0.001, respectively). In contrast, the CHA of IL-6 was significantly higher in the PMMA-CRRT group than in the AN69ST-CRRT group (*P* < 0.001). In addition, the 28-day mortality was not significantly different between the two groups (50% in AN69ST-CRRT vs. 30.8% in PMMA-CRRT,* P* = 0.26).

**Conclusion:**

AN69ST and PMMA membranes have different cytokine CHA in patients with sepsis. Therefore, these two hemofilters may have to be used depending on the target cytokine.

*Trial registration number:* This study was registered in the University Hospital Medical Information Network on November 1, 2017 (Trial No: UMIN000029450, https://center6.umin.ac.jp).

**Supplementary Information:**

The online version contains supplementary material available at 10.1186/s40001-023-01184-6.

## Background

Sepsis, which is a life-threatening organ dysfunction, is caused by the dysregulated host response to infection [1]. High blood levels of proinflammatory and anti-inflammatory cytokines are associated with mortality [2]. This signal activates leukocytes and induces the synthesis of pro- and anti-inflammatory cytokines, including tumor necrosis factor-alpha (TNF-α), interleukin (IL)-1, IL-6, IL-8, and IL-10. In addition, the massive release of cytokines in the blood has been described as a “cytokine storm” and is believed to be responsible for major organ dysfunction [3]. Moreover, cytokine adsorption therapy may restore the balance of proinflammatory and anti-inflammatory mediator responses in patients with sepsis [4]. Thus, cytokine adsorption therapy may reduce the mortality rate; however, this potential has not yet been proven.

Acute kidney injury (AKI) is significantly associated with a high mortality rate in critically ill patients [5]. Continuous renal replacement therapy (CRRT) is widely used in the intensive care unit (ICU) [6]. In addition, cytokine-adsorbing hemofilters, including polyethyleneimine (PEI)-coated polyacrylonitrile (AN69ST) or polymethylmethacrylate (PMMA) [7–9] membranes, are commonly used in Japan for appropriate control of cytokine overproduction in patients with sepsis. In vitro studies have demonstrated that AN69ST membranes have a superior adsorption ability for TNF-α, IL-8 [10], and high-mobility group box 1 (HMGB1) [11], whereas PMMA membranes strongly adsorb IL-6 [10]. A previous observational study showed that the ability of adsorption for chemokines was different between the AN69ST and PMMA membranes [12]. However, clinical evidence comparing cytokine adsorption in these two hemofilters is lacking, and from these aspects, we evaluated the cytokines, including TNF-α, IL-6, IL-8, IL-10, IL-18, monokine induced by interferon-γ (MIG), macrophage inflammatory protein (MIP)-1α, and HMGB1 in the present study. This open-label randomized controlled trial (RCT) aimed to clarify the difference in cytokine adsorption ability between the AN69ST and PMMA hemofilters.

## Methods

### Trial design and patients

This pilot open-label RCT was conducted at the Tertiary Emergency and Critical Care Center of Fukuoka University Hospital (Fukuoka, Japan) according to the Declaration of Helsinki. Our emergency and closed ICU has 32 beds. The trial was registered at the University Hospital Medical Information Network (UMIN000029450), and its protocol was previously published (https://center6.umin.ac.jp). Eligible patients (at least 18 years of age) included those (1) with sepsis diagnosed using the Sepsis-3 definition [1] on admission; (2) who underwent CRRT therapy; and (3) with AKI diagnosed according to the Kidney Disease: Improving Global Outcome [13] criteria or had undergone prior dialysis for treating end-stage kidney disease (EDKD). Sepsis was defined as cases caused not only by bacterial, but also by viral infection [1]. Therefore, patients with coronavirus disease 2019 (COVID-19) were subjected to the same eligibility criteria, randomization procedure, consent process, and interventions as other patients with sepsis. Patients who had COVID-19 included those with AKI and without AKI, as the Japanese Ministry of Health, Labour, and Welfare recommends considering CRRT for patients with COVID-19 (https://www.mhlw.go.jp/content/000936655.pdf). Regarding the payment for renal replacement therapy (RRT), Japan has adopted a universal health insurance system. Therefore, patients’ financial condition does not influence physicians’ decisions about medical interventions and RRT induction [14]. Furthermore, no evidence regarding the optimal RRT conditions for AKI is firmly established [15]. Therefore, the timing of RRT initiation depends on the attending clinician’s decision.

### Interventions and procedures

The PMMA and AN69ST groups were defined based on the PMMA and AN69ST membranes. CRRT was initiated immediately after ICU admission. The patients were randomly assigned (1:1) to the AN69ST or PMMA groups. Random numbers were generated using the Excel random function (Microsoft Japan Co., Ltd., Tokyo, Japan), and patients were then randomly assigned to groups according to the hemofilter used.

All patients were randomized immediately following ICU admission, and CRRT was initiated in the ICU. CRRT was performed using ACH-10® or ACH-Σ® (Asahi Kasei Medical Co., Ltd., Tokyo, Japan). The hemofilters used were an AN69ST (sepXiris150; Baxter Co. Ltd., Tokyo, Japan) or a PMMA membrane (Hemofeel CH1.5N; Toray Medical Co., Ltd., Tokyo, Japan). All CRRT modes were continuous hemodiafiltration. The operating conditions were as follows: quantity of blood flow (QB), 80–100 mL/min; dialysate flow rate, 500 mL/h; and filtration flow rate (QF), 300 mL/h. Sublood-BS (Fuso Pharmaceutical Industries, Osaka, Japan) was used as both the dialysate and replacement fluid. Nafamostat mesylate (NM) (Asahi Kasei Pharma Corp., Tokyo, Japan) was administered as an anticoagulant, and the dose was maintained in the range of 0–30 mg/h. The activated clotting time after hemofiltration was maintained at > 180 s, and it was measured using the Hemochron Response (Heiwa Bussan, Co. Ltd., Tokyo, Japan). NM is a protease inhibitor that strongly inhibits the activity of various coagulation enzymes [16]. Because of its short half-life, NM is regarded as a useful regional anticoagulant during hemodialysis in patients with bleeding tendencies [17]. Accordingly, NM has been in use since 1990 (primarily in Japan) [18] as a regional and widely used as anticoagulant during blood purification in Japanese ICUs [19].

### Data and sample collection

The baseline data, including patients’ characteristics such as age, sex, comorbidities, AKI stage on admission, source of infection, and detected microorganism (COVID-19 was diagnosed based on the detection of severe acute respiratory syndrome coronavirus 2 [SARS-CoV-2] on reverse transcription-polymerase chain reaction or SARS-CoV-2 antigen from a nasopharyngeal swab sample), diagnosed septic shock [1] on admission; laboratory data, including white blood cell and platelet counts and total bilirubin, creatinine, lactate, C-reactive protein, and procalcitonin levels; whether or not mechanical ventilation was performed; partial pressure of arterial oxygen/fraction of inspired oxygen (PaO_2_/F_I_O_2_) ratio; vasopressor index (VAI) [20]; and Acute Physiology and Chronic Health Evaluation II [21] and Sequential Organ Failure Assessment (SOFA) [22] scores on admission, hemofilter lifetime, the number of hemofilters used within 24 h after CRRT initiation, and sample collection time after CRRT initiation were also collected.

Blood samples and filtrates were drawn from the extracorporeal circuit at the inlet and outlet of the hemofilter 2–6 and 12–24 h after initiating CRRT (circuit schema is shown in Additional file 1) to evaluate cytokine clearance of hemofilter adsorption (CHA), and blood samples were collected before ICU admission and on days 2–4 and 5–7 after ICU admission from the peripheral arterial catheter. Whole blood was collected with ethylenediaminetetraacetic acid-2 K as an anticoagulant in a conventional blood collection tube NP-EN0557-6 (NIPRO Co., Osaka, Japan). The blood was centrifuged at 1,400 × g for 15 min, and the plasma was stored at -80 °C until measurement.

TNF-α, IL-1β, IL-6, IL-8, IL-10, MIG, and MIP-1α levels were measured using a HISCL-5000 (Sysmex Co., Kobe, Japan) [23] and HMGB1 enzyme-linked immunosorbent assay kit (Shino-Test Corp., Tokyo Japan).

### Outcomes

The primary outcome was cytokine CHA. Plasma cytokine clearance was calculated according to the following formula [8, 24]:

$${\text{Plasma clearance}};{\text{ CLs }} = \, ({\text{CBi}} - {\text{CBo}})/{\text{CBi}} \times \left( {{\text{QB}} - {\text{QF}}} \right) + {\text{QF}},$$

$${\text{Transmembrane clearance}};{\text{ FLs }} = \, \left( {{\text{CF}}/{\text{CBi}}} \right) \, \times {\text{ QF,}}$$

$${\text{Clearance of hemofilter adsorption CHA }} = {\text{ CLs}} - {\text{FLs,}}$$

where CBi is the blood cytokine level at the filter inlet, CBo represents the blood cytokine level at the filter outlet, QB is the quantity of blood flow (mL/min), QF is the ultrafiltrate flow rate (mL/h), and CF is the cytokine level in the filtrate.

Secondary endpoints included blood cytokine levels upon admission to the ICU and on days 2–4 and 5–7 after ICU admission, ICU mortality, 28-day all-cause mortality, VAI [20], PaO_2_/F_I_O_2_ ratio at 48 h following the CRRT procedure, and ICU-free days (ICUFDs). The VAI was calculated as follows; (dopamine dose × 1) + (dobutamine dose × 1) + adrenaline dose × 100) + (noradrenaline dose × 100), with all doses expressed as μg/kg/min. ICUFDs were calculated as follows: ICUFDs = 0 if the patient died within the first 28 days; ICUFDs = (28-*x*) if the patient survived for more than 28 days, where *x* is the number of days spent in the ICU; and ICUFDs = (28-*y*) if the patient was transferred to another hospital before 28 days had elapsed, where *y* is the number of days spent in the ICU.

Safety and feasibility outcomes included the number of patients with serious adverse events and reactions in both arms.

### Statistical analyses

Data are presented as medians (interquartile ranges) for continuous variables and percentages for categorical variables. We used the Wilcoxon, Steel–Dwass, and Chi-square or Fisher’s exact tests for comparing two groups of continuous variables, multiple comparisons between continuous variables, and comparing categorical variables, respectively. Furthermore, ICU and 28-day mortality rates were analyzed using multivariate logistic regression, and the explanatory variables were age and SOFA score. The data were analyzed using the statistical software JMP12 for Windows (SAS Institute Japan, Tokyo, Japan). Results were considered statistically significant at P-values less than 0.05. Since this was a pilot study, a sample size estimation was not conducted.

## Results

### Patient baseline characteristics

**Fig. 1:**
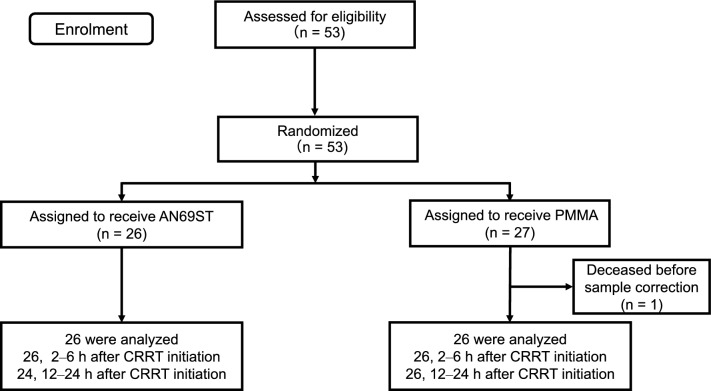
Flowchart of trial patients. *AN69ST* polyethyleneimine-coated polyacrylonitrile, *PMMA* polymethylmethacrylate, *CRRT* continuous renal replacement therapy

Overall, 53 patients were enrolled and randomized to either the AN69ST (*n* = 26) or PMMA (*n* = 27) group. One patient was excluded because of death before sample collection. Therefore, 26 patients from each group were included in the primary and secondary analyses (Fig. 1). Baseline characteristics including age, sex, comorbidity, AKI severity, source of infection, detected microorganism, laboratory findings, treatment, and cytokine levels (at the inlet of the hemofilter, 2–6 h after initiating CRRT), did not differ between the groups. All non-AKI patients had COVID-19-related sepsis. In contrast, the SOFA score was significantly higher in the AN69ST group than in the PMMA group (*P* < 0.01) (Table 1).

**Table 1:** Patient characteristics

Variable	AN69ST	PMMA	*P* value^a^
	(*n* = 26)	(*n* = 26)	
Age, years	69.5 (63.3–74.0)	71.5 (63.3–77.3)	0.83
Sex, male	19 (73.0)	19 (73.0)	1.00
Comorbidity			
Hypertension	12 (46.2)	13 (50.0)	0.78
Diabetes	9 (34.6)	8 (30.8)	0.77
Chronic heart failure	2 (7.7)	0 (0.0)	0.24
Coronary artery disease	3 (11.5)	1 (3.8)	0.61
Chronic obstructive pulmonary disease	1 (3.8)	2 (7.7)	1.00
Chronic liver disease	0 (0.0)	0 (0.0)	
KDIGO stage			0.76
Non-AKI^b^	4 (15.4)	3 (11.5)	
Stage 1	7 (26.9)	8 (30.8)	
Stage 2	3 (11.5)	3 (11.5)	
Stage 3	12 (46.2)	12 (46.2)	
Prior dialysis due to ESKD	6 (23.1)	4 (15.4)	0.50
Septic shock	6 (23.1)	5 (19.2)	0.74
Source of infection			0.96
Respiratory	13 (50.0)	12 (46.2)	
Intraabdominal	8 (30.8)	10 (38.5)	
Skin and soft tissue	3 (11.5)	2 (7.7)	
Urinary	0 (0)	0 (0.0)	
Others	2 (7.7)	2 (7.7)	
Microorganisms isolated/or positively identified			
G( +)	2 (7.7)	5 (19.2)	0.42
G(−)	4 (15.4)	4 (15.4)	1.00
G( +) and G(−)	8 (30.8)	2 (7.7)	0.08
SARS-CoV-2	7 (26.9)	7 (26.9)	1.00
Others	5 (19.2)	3 (11.5)	0.70
Not detected and unknown	4 (15.4)	6 (23.1)	0.73
Laboratory test results on admission			
WBC, × 10^9^ counts/L	9.4 (6.6–14.3)	10.3 (7.6–17.5)	0.39
Plt, × 10^9^ counts/L	26.9 (10.2–115.3)	47.4 (15.4–152.5)	0.44
T-bil, mg/dL	1.1 (0.6–2.6)	0.8 (0.6–1.3)	0.18
Cr, mg/dL	2.1 (1.5–2.9)	2.6 (1.0–5.2)	0.67
Lac, mg/dL	26.0 (13.8–61.5)	14.7 (11.2–33.6)	0.09
CRP, mg/dL	10.0 (7.2–18.8)	10.8 (4.4–21.2)	0.84
PCT, ng/mL	2.7 (1.3–10.9)	7.0 (0.3–45.1)	0.88
Mechanical ventilation	22 (84.6)	22 (84.6)	1.00
PaO_2_/F_I_O_2_ ratio	134.3 (88.8–315.8)	145.5 (102.2–249.0)	0.73
VAI	17.9 (5.5–35.2)	6.1 (0–31.1)	0.07
APACHE II score	26.0 (18.3–28.8)	19.5 (18.0–24.8)	0.09
SOFA score	12.0 (10.3–15.0)	8.5 (7.0–12.8)	< 0.01
Anticoagulant used for CRRT			1.00
Heparin	2 (7.7)	1 (3.8)	
Nafamostat mesylate	24 (92.3)	25 (96.2)	
Baseline cytokine levels^c^			
HMGB1, ng/mL	3.6 (2.0–5.5)	3.1 (1.9–6.6)	0.88
TNF-α, pg/mL	2.9 (1.3–6.7)	2.8 (1.3–5.6)	0.79
IL-6, pg/mL	1736.2 (584.0–16,407.0)	1369.4 (303.4–3791.8)	0.48
IL-8, pg/mL	73.2 (26.8–518.2)	61.4 (34.2–357.1)	0.80
IL-10, pg/mL	34.1 (14.0–142.1)	47.1 (25.9–149.9)	0.24
IL-18, pg/mL	868.6 (553.1–1286.8)	637.6 (516.5–1390.2)	0.62
MIG, pg/mL	76.8 (46.9–238.8)	107.1 (41.7–438.6)	0.52
MIP-1α, pg/mL	146.5 (72.2–323.8)	149.6 (65.2–242.3)	0.60
CRRT prescribed			
Blood flow rate, mL/min	80 (80–80)	80 (80–80)	0.57
Ultrafiltrate flow rate, mL/h	300 (300–318)	300 (300–314)	0.71
Dialysate flow rate, mL/h	500 (500–500)	500 (500–500)	0.23
CRRT filter life time			
1st filter, h	17.0 (8.7–22.8)	13.3 (4.1–27.7)	0.66
2nd filter, h	9.7 (3.2–18.3)	12.6 (3.5–21.3)	0.43
Number of filter exchanges within 24 h	1 (0–1.3)	1 (0–1)	0.72
Time window within which blood samples and filtrates were drawn			
2–6 h, h	2.2 (2.0–3.0)	2.3 (2.0–3.3)	0.89
12–24 h, h	14.8 (13.7–18.4)	14.7 (12.4–19.2)	0.98

Data are given as medians and interquartile ranges or *n* (%)

*AN69ST* polyethyleneimine-coated polyacrylonitrile, *PMMA* polymethylmethacrylate, *IQR* interquartile range, *KDIGO* Kidney Disease: Improving Global Outcomes, *ESKD* end-stage renal failure, *AKI* acute kidney injury, *G( +)* Gram-positive infection, *G(−)* Gram-negative infection, *SARS-CoV-2* severe acute respiratory syndrome coronavirus 2, *WBC* white blood cell, *Plt* platelet, *T-bil* total bilirubin, *Cr* creatinine, *Lac* serum lactic acid, *CRP* C-reactive protein, *PCT* procalcitonin, *PaO*_*2*_*/F*_*I*_*O*_*2*_ partial pressure of arterial oxygen/fraction of inspired oxygen, *VAI* vasopressor index, *APACHE* acute physiology and chronic health evaluation, *SOFA* sequential organ failure assessment, *CRRT* continuous renal replacement therapy, *HMGB1* high-mobility group box 1, *TNF* tumor necrosis factor, *IL* interleukin, *MIG* monokine induced by interferon-γ, *MIP-1α* macrophage inflammatory protein 1 alpha

^a^Wilcoxon test or *χ*^2^ test

^b^All non-AKI patients had coronavirus disease 2019-related sepsis

^c^Samples were at the inlet of the hemofilter 2–6 h after initiating CRRT

### Primary outcomes

A comparison of cytokine CHA is presented in Table 2. The ability of CHA of HMGB1, TNF-α, IL-8, MIG, and MIP-1α was significantly higher in the AN69ST group than in the PMMA group. In contrast, PMMA membranes had a significantly higher ability to adsorb IL-6 than AN69ST membranes. Cytokine levels at each sampling point are presented in Additional file 2.

**Table 2:** Primary outcome (cytokine clearance of hemofilter adsorption)

Mediators	Clearance	Sampling time window	AN69ST	PMMA	*P* value^a^
			(*n* = 26)	(*n* = 26)	
HMGB1	Plasma clearance (mL/min)	2–6 h	43.6 (30.2–52.8)	5.1 (− 15.5–17.5)	< 0.001
		12–24 h	20.1 (6.7–45.2)	0.9 (− 20.0–11.1)	< 0.001
	Transmembrane clearance (mL/min)	2–6 h	0 (0–0)	0 (0–0.34)	0.16
		12–24 h	0 (0–0.35)	0 (0–0.18)	0.47
	Clearance of hemofilter adsorption (mL/min)	2–6 h	43.6 (29.8–52.8)	3.6 (-15.9–17.5)	< 0.001
		12–24 h	20.1 (6.2–45.2)	− 0.7 (− 20.0–11.1)	< 0.001
TNF-α	Plasma clearance (mL/min)	2–6 h	33.5 (28.8–36.3)	26.2 (7.5–30.0)	< 0.01
		12–24 h	24.7 (18.6–32.3)	13.8 (9.2–24.1)	< 0.01
	Transmembrane clearance (mL/min)	2–6 h	0.01 (0–0.07)	0 (0–0.06)	0.70
		12–24 h	0.04 (0–0.09)	0 (0–0.02)	< 0.05
	Clearance of hemofilter adsorption (mL/min)	2–6 h	33.5 (29.4–36.8)	26.4 (6.9–30.2)	< 0.01
		12–24 h	25.0 (20.4–32.2)	14.0 (9.1–24.9)	< 0.01
IL-6	Plasma clearance (mL/min)	2–6 h	9.4 (7.8–12.1)	17.6 (10.7–22.5)	< 0.001
		12–24 h	9.2 (5.6–12.0)	9.1 (4.0–14.7)	0.82
	Transmembrane clearance (mL/min)	2–6 h	1.8 (1.6–2.2)	0 (0–0.01)	< 0.001
		12–24 h	1.72 (1.26–2.12)	0.08 (0–0.22)	< 0.01
	Clearance of hemofilter adsorption (mL/min)	2–6 h	7.6 (4.3–10.6)	17.6 (10.7–22.4)	< 0.001
		12–24 h	7.4 (3.4–9.8)	9.1 (3.7–14.6)	0.29
IL-8	Plasma clearance (mL/min)	2–6 h	47.4 (33.0–50.5)	6.2 (− 8.7–12.0)	< 0.01
		12–24 h	33.9 (14.9–46.4)	3.4 (− 31.0–9.3)	< 0.01
	Transmembrane clearance (mL/min)	2–6 h	0.15 (0.04–0.87)	1.34 (1.04–4.89)	< 0.01
		12–24 h	0.4 (0.2–1.2)	3.9 (3.1–11.2)	< 0.01
	Clearance of hemofilter adsorption (mL/min)	2–6 h	47.0 (31.8–50.7)	4.5 (-11.4–10.6)	< 0.001
		12–24 h	34.1 (13.6–44.6)	0.7 (− 50.0–5.8)	< 0.001
IL-10	Plasma clearance (mL/min)	2–6 h	28.3 (23.1–36.6)	27.2 (16.5–30.3)	0.37
		12–24 h	26.0 (18.1–29.1)	18.8 (12.9–24.8)	0.07
	Transmembrane clearance (mL/min)	2–6 h	0 (0–0.01)	0 (0–0)	0.18
		12–24 h	0.02 (0.01–0.06)	0.01 (0–0.01)	< 0.01
	Clearance of hemofilter adsorption (mL/min)	2–6 h	28.3 (23.1–35.6)	27.2 (16.5–30.3)	0.36
		12–24 h	26.0 (17.7–29.0)	18.7 (12.9–24.8)	0.07
IL-18	Plasma clearance (mL/min)	2–6 h	− 0.4 (-3.3–2.3)	− 0.6 (− 3.0–1.5)	0.73
		12–24 h	− 0.2 (-3.1–2.4)	− 2.5 (− 4.7–0.7)	0.17
	Transmembrane clearance (mL/min)	2–6 h	0.01 (0–0.01)	0.10 (0.06–0.13)	< 0.001
		12–24 h	0.01 (0–0.01)	0.01 (0.04–0.01)	< 0.001
	Clearance of hemofilter adsorption (mL/min)	2–6 h	− 0.4 (-3.3–2.3)	− 0.7 (− 3.2–1.4)	0.62
		12–24 h	− 0.6 (− 2.4–2.5)	− 2.5 (− 4.7–0.6)	0.09
MIG	Plasma clearance (mL/min)	2–6 h	64.9 (61.9–66.9)	24.0 (18.8–32.9)	< 0.001
		12–24 h	59.3 (52.4–61.8)	10.7 (7.4–19.2)	< 0.001
	Transmembrane clearance (mL/min)	2–6 h	0.13 (0.06–0.20)	0.19 (0.05–0.76)	0.30
		12–24 h	0.48 (0.18–0.86)	2.00 (0.66–2.33)	< 0.05
	Clearance of hemofilter adsorption (mL/min)	2–6 h	64.6 (61.6–66.7)	24.0 (18.0–32.2)	< 0.001
		12–24 h	58.6 (49.2–61.2)	8.3 (5.2–17.6)	< 0.001
MIP-1α	Plasma clearance (mL/min)	2–6 h	64.3 (60.9–65.9)	38.9 (16.1–45.7)	< 0.001
		12–24 h	54.9 (48.1–58.9)	20.9 (5.0–31.7)	< 0.001
	Transmembrane clearance (mL/min)	2–6 h	0.02 (0.01–0.05)	0.01 (0–0.02)	< 0.05
		12–24 h	0.22 (0.04–0.67)	0.14 (0.01–0.61)	0.36
	Clearance of hemofilter adsorption (mL/min)	2–6 h	64.2 (60.9–65.9)	38.9 (16.0–45.8)	< 0.001
		12–24 h	54.8 (47.4–58.9)	20.6 (4.3–31.6)	< 0.001

Data are given as medians and interquartile ranges

*CBi* blood cytokine level at the filter inlet, *CBo* blood cytokine level at the outlet, *QB* blood flow rate (mL/min), *QF* flow rate of the ultrafiltrate, *CF* cytokine level in the filtrate

*AN69ST* polyethyleneimine-coated polyacrylonitrile, *PMMA* polymethylmethacrylate, *HMGB1* high-mobility group box 1, *TNF* tumor necrosis factor, *IL* interleukin, *MIG* monokine induced by interferon-γ, *MIP-1α* macrophage inflammatory protein 1 alpha

^a^Wilcoxon test

$${\text{Plasma clearance }} = \, ({\text{CBi}} - {\text{CBo}})/{\text{CBi}} \times \left( {{\text{QB}} - {\text{QF}}} \right) + {\text{QF}},$$

$${\text{Transmembrane clearance }} = \, \left( {{\text{CF}}/{\text{CBi}}} \right) \, \times {\text{ QF,}}$$

$${\text{Clearance of hemofilter adsorption }} = {\text{ plasma clearance}} - {\text{transmembrane clearance}}{.}$$

### Secondary outcomes

**Fig. 2:**
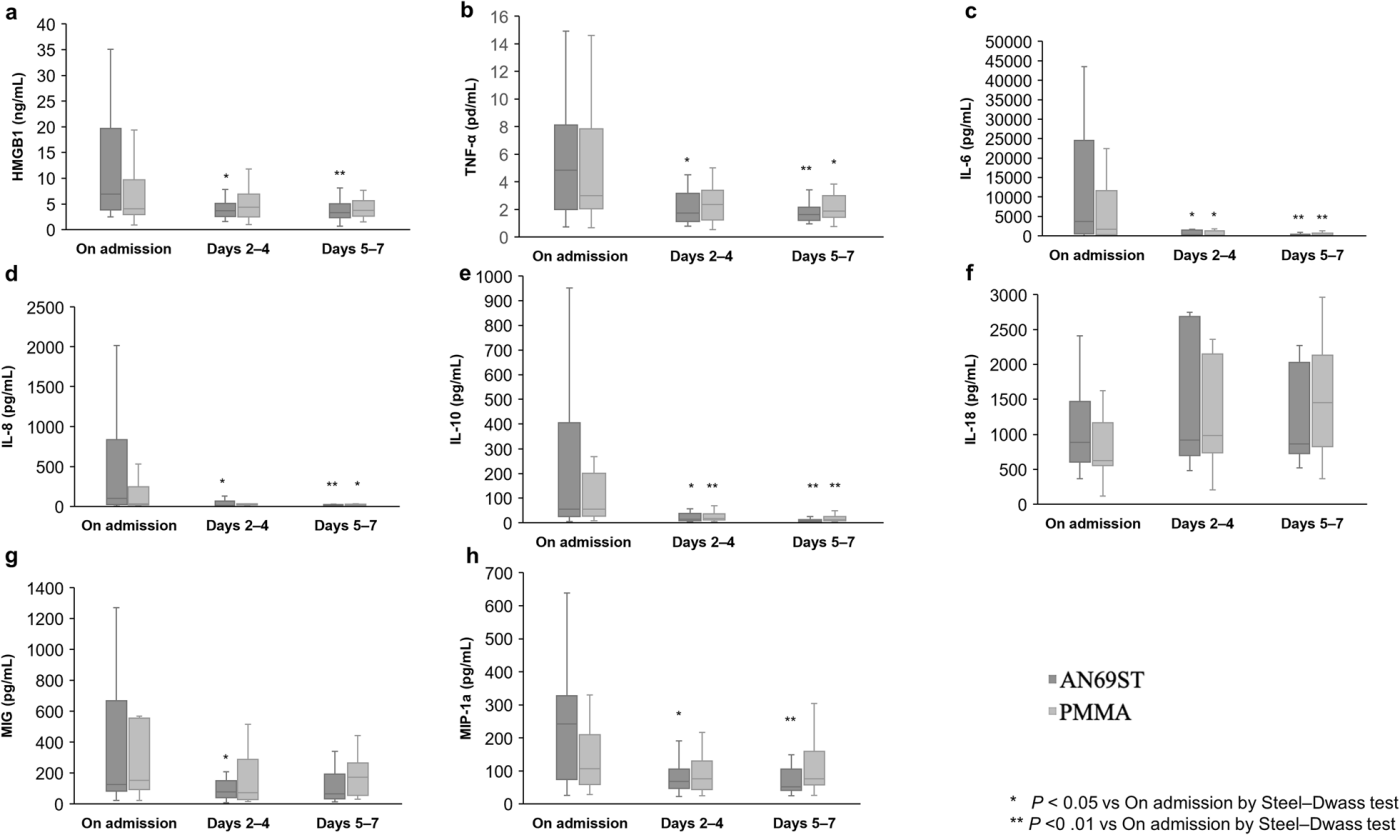
Time course of cytokine levels. The Kruskal–Wallis test followed by the Steel–Dwass test were used to compare time courses. The Wilcoxon test was used to compare the AN69ST- and PMMA-CRRT groups at each time point. No significant differences between the two groups were found at any time point. **a**: HMGB1, **b**: TNF-α, **c**: IL-6, **d**: IL-8, **e**: IL-10, **f**: IL-18, **g**: MIG, and **h**: MIP-1a. *AN69ST* polyethyleneimine-coated polyacrylonitrile, *PMMA* polymethylmethacrylate, *CRRT* continuous renal replacement therapy; *HMGB-1* high-mobility group box 1, *TNF-α* tumor necrosis factor, *IL* interleukin, *MIG* monokine induced by interferon-γ, *MIP* macrophage inflammatory protein

The time course of cytokine levels within 7 days of admission is shown in Fig. 2. In the AN69ST group, HMGB1, TNF-α, IL-6, IL-8, IL-10, MIG, and MIP-1α levels were significantly decreased; in the PMMA group, TNF-α, IL-6, IL-8, and IL-10 levels were significantly decreased. No significant difference was observed in all cytokine levels between the AN69ST and PMMA groups at each timepoint.

Furthermore, ICU and 28-day all-cause mortalities were not significantly different between the two groups in the unadjusted (odds ratio [OR] 1.89, 95% confidence interval [CI] 0.62–5.76 for ICU mortality and OR 2.25, 95% CI 0.72–7.00 for 28-day all-cause mortality) and adjusted analyses (OR 1.65, 95% CI 0.49–5.90 for ICU mortality and OR 2.34, 95% CI 0.67–8.70 for 28-day all-cause mortality) (Table 3).

**Table 3:** Secondary outcomes

Outcome	AN69ST (*n* = 26)	PMMA (*n* = 26)	AN69ST vs. PMMA			
			Unadjusted 95% CI	Adjusted ^a^ 95% CI	Unadjusted *P* value	Adjusted ^a^ *P* value
ICU mortality, n (%)	13 (50.0)	9 (34.6)	1.89 (0.62–5.76)	1.65 (0.48–5.85)	0.40	0.42
28-day all-cause mortality, n (%)	13 (50.0)	8 (30.8)	2.00 (0.67–6.23)	2.34 (0.67–8.72)	0.22	0.18
ICUFDs, median (IQR)	0 (0–18.3)	0 (0–16.0)	0.99 (0.93–1.05)	–	0.65	–
VAI at 48 h after CRRT initiation, median (IQR)	0 (0–8.4)	0 (0–15.0)	1.01 (0.99–1.05)	–	0.32	–
P/F ratio at 48 h after CRRT initiation, median (IQR)	220 (61.4–321.0)	227 (91.5–308)	1.00 (0.99–1.00)	–	0.71	–

*AN69ST* polyethyleneimine-coated polyacrylonitrile, *PMMA* polymethylmethacrylate, *CI* confidence interval, *ICU* intensive care unit, *ICUFDs* ICU-free days, *IQR* interquartile range; *CRRT* continuous renal replacement therapy, *VAI* vasopressor index, *P/F* partial pressure of arterial oxygen/fraction of inspired oxygen

^a^Analyzed by multilogistic regression model and explanatory variables such as age and SOFA score

### Safety and feasibility outcomes

No serious adverse events were observed in either group (Additional file 3).

## Discussion

To the best of our knowledge, this study is the first RCT to evaluate the difference in cytokine CHA between the AN69ST and PMMA hemofilters in a clinical setting. We found that AN69ST and PMMA membranes had significantly different cytokine CHA in patients with sepsis in different time points at 2–4 h and 12–24 h after CRRT initiation (Table 2).

The AN69 membrane is an electronegative copolymer of acrylonitrile and sodium methanesulfonate. AN69 can undergo adsorption in the membrane bulk through electrostatic interaction. In contrast, AN69ST was achieved by neutralizing the surface in contact with blood by ionic grafting of a polycationic polymer in AN69; however, AN69ST can also be adsorbed in the membrane bulk through electrostatic interaction [25]. The AN69ST group showed significantly superior ability to adsorb HMGB1, MIG, and MIP-1α compared with the PMMA group (Table 2). HMGB1 is well known to be adsorbed by AN69ST membranes in vitro [11, 26] and as a damage-associated molecular pattern. HMGB1 inhibitors have potential therapeutic applications [27, 28]. Moreover, MIG and MIP-1α are known as chemokines, which are drivers of cytokine storms due to infection [29]. AN69ST membranes reportedly have a higher chemokine adsorption ability than PMMA membranes, as evaluated using time-of-flight or mass spectrometry analysis [12].

An in vitro closed-loop circulation system study showed that time-dependent changes of transmembrane pressure (TMP) were not observed but time-dependent superiority for CHA ability was observed in AN69ST membrane in comparison with PMMA for HMGB1 [11], possibly because AN69ST can adsorb mediators not only on the surface, but also in the bulk of the membrane with hydrophobic bonding [11, 12]. In the present study, the ability of CHA was superior not only 2–6 h after CRRT initiation but also 12–24 h after CRRT initiation in AN69ST rather than PMMA, which supports the findings of Yumoto et al. [11] even though in a clinical setting. Because AN69ST is electronegative, positively charged mediators such as TNF-α [10], IL-8 [10], or NM [30] were adsorbed more than other membranes. Moriyama et al. [10] reported that different pH solutions with dissolved TNF-α, IL-6, and IL-8 were closed-loop circuit system in vitro, thus the pH of the test solution shifted from 7.6 to 6.8, the CLs of TNF-α, IL6, and IL-8 increased in the AN69ST hemofilter; whereas, no such trend was observed in the PMMA hemofilter. These results indicated the involvement of ionic interactions in cytokine adsorption by the AN69ST membrane but not the PMMA membrane. The present study also found that the CHA of TNF-α and IL-8 was superior in AN69ST, compared to PMMA. Isoelectric points and molecular weights of cytokine are shown in Additional file 4. IL-10 and MIG are more positively charged than TNF-α; however, IL-18, HMGB1, and MIP-1a are more negatively charged than TNF-α; therefore, further analysis is warranted for CHA mechanism in AN69ST membrane.

In contrast, PMMA membranes have a higher CHA ability for IL-6 than for AN69ST membranes (Table 2). Furthermore, the time course of IL-6 levels was significantly decreased in the PMMA group. IL-6 is a well-known sepsis biomarker, and its levels correlate with the severity of sepsis [31]. Blockade therapy is beneficial for cytokine storms [32]. Based on this study’s findings, we may have to distinguish between AN69ST and PMMA membrane use depending on the target molecules.

Cytokine levels were significantly decreased in both the AN69ST and PMMA groups (Fig. 2). In the AN69ST group, HMGB1, MIG, and MIP-1α levels were significantly decreased after ICU admission, but this was not observed in the PMMA group. However, no significant difference was observed in the cytokine levels between the two groups in terms of baseline characteristics (Table 1). Moreover, the baseline SOFA score was significantly higher in the AN69ST group than in the PMMA group; however, regarding the secondary endpoints, no significant difference was observed in clinical benefit after adjustment for the baseline SOFA score (Table 3). The present study was pilot study; therefore, the sample size was too small, indicating the need for further studies.

Some observational studies [33–35] have shown that AN69ST hemofilters are superior to non-AN69ST hemofilters. Furthermore, AN69ST and PMMA membranes have already been widely used in Japan [33–37], and no serious adverse events were observed in either group. Therefore, future RCTs are warranted to investigate the effect of AN69ST and PMMA hemofilters on clinical outcomes.

### Strengths and limitations

The obvious strength of our study is the use of randomization to minimize selection bias. However, this study has some limitations. First, blinding of the interventions was not performed. Second, because this was a pilot, single-center study, generalizability is insufficient. Third, the present study did not have a control group that was not treated with CRRT. Therefore, this study did not provide information about endogenous clearance rates in septic patients, indicating that part of the decreased cytokine levels in blood may not depend on CRRT. Fourth, the sampling time windows (2–6 h and 12–24 h) were relatively wide. However, no significant differences in sampling time windows were observed between the two groups (Table 1), and even after excluding patients with a circuit life span of within 24 h, CHA ability was not different from the CHA ability when including all patients (Additional file 5).

## Conclusions

Our first pilot RCT showed that AN69ST and PMMA hemofilters have different cytokine CHA ability in patients with sepsis. However, no significant difference was observed in the present pilot clinical study. Therefore, these two hemofilters may have to be used depending on the target cytokine.

## Supplementary Information

**Additional file 1.** Circuit schema.

**Additional file 2.** Cytokine levels at each sampling point.

**Additional file 3.** Number of serious adverse events.

**Additional file 4.** Molecular weights and isoelectric points of cytokines**.** The theoretical molecular weights and isoelectric points of each cytokine are indicated in the Table and Figure***. Values were calculated by Expasy (https://web.expasy.org/compute_pi/) based on the amino acid sequence of matured protein. *HMGB-1* high-mobility group box 1, *TNF-α* tumor necrosis factor, *IL* interleukin, *MIG* monokine induced by interferon-γ, *MIP* macrophage inflammatory protein.

**Additional file 5.** Results of primary endpoint analysis after excluding patients with circuit life span of within 24 h.

## Data Availability

The data that support the findings of this study are available from authors, but restrictions apply to the availability of these data, which were used under license for the current study, and so are not publicly available. Data are however available from the authors upon reasonable request and with permission of the Medical Ethics Review Board of Fukuoka University.

## Abbreviations

AKI: Acute kidney injury
CHA: Clearance of hemofilter adsorption
CI: Confidence interval
CRRT: Continuous renal replacement therapy
COVID-19: Coronavirus disease 2019
QF: Filtration flow rate
HMGB1: High-mobility group box 1
ICUFDs: Intensive care unit-free days
ICU: Intensive care unit
IL: Interleukin
MIP: Macrophage inflammatory protein
MIG: Monokine induced by interferon-γ
NM: Nafamostat mesylate
OR: Odds ratio
PaO_2_/F_I_O_2_: Partial pressure of arterial oxygen/fraction of inspired oxygen
AN69ST: Polyacrylonitrile
PEI: Polyethyleneimine
PMMA: Polymethylmethacrylate
QB: Quantity of blood flow
RCT: Randomized controlled trial
RRT: Renal replacement therapy
SOFA: Sequential Organ Failure Assessment
SARS-CoV-2: Severe acute respiratory syndrome coronavirus 2
TMP: Transmembrane pressure
TNF-α: Tumor necrosis factor-alpha
VAI: Vasopressor index
