# Supplementary material for: Comparison of the cytokine adsorption ability in continuous renal replacement therapy using polyethyleneimine-coated polyacrylonitrile (AN69ST) or polymethylmethacrylate (PMMA) hemofilters: a pilot single-center open-label randomized control trial
Source: Eur J Med Res. 2023 Jun 30;28:208. doi: 10.1186/s40001-023-01184-6 (PMC10314474; doi:10.1186/s40001-023-01184-6)
Supplement: Supplementary file 1 — Additional file 1. Circuit schema. [file 40001_2023_1184_MOESM1_ESM.pptx]

## Slide 1
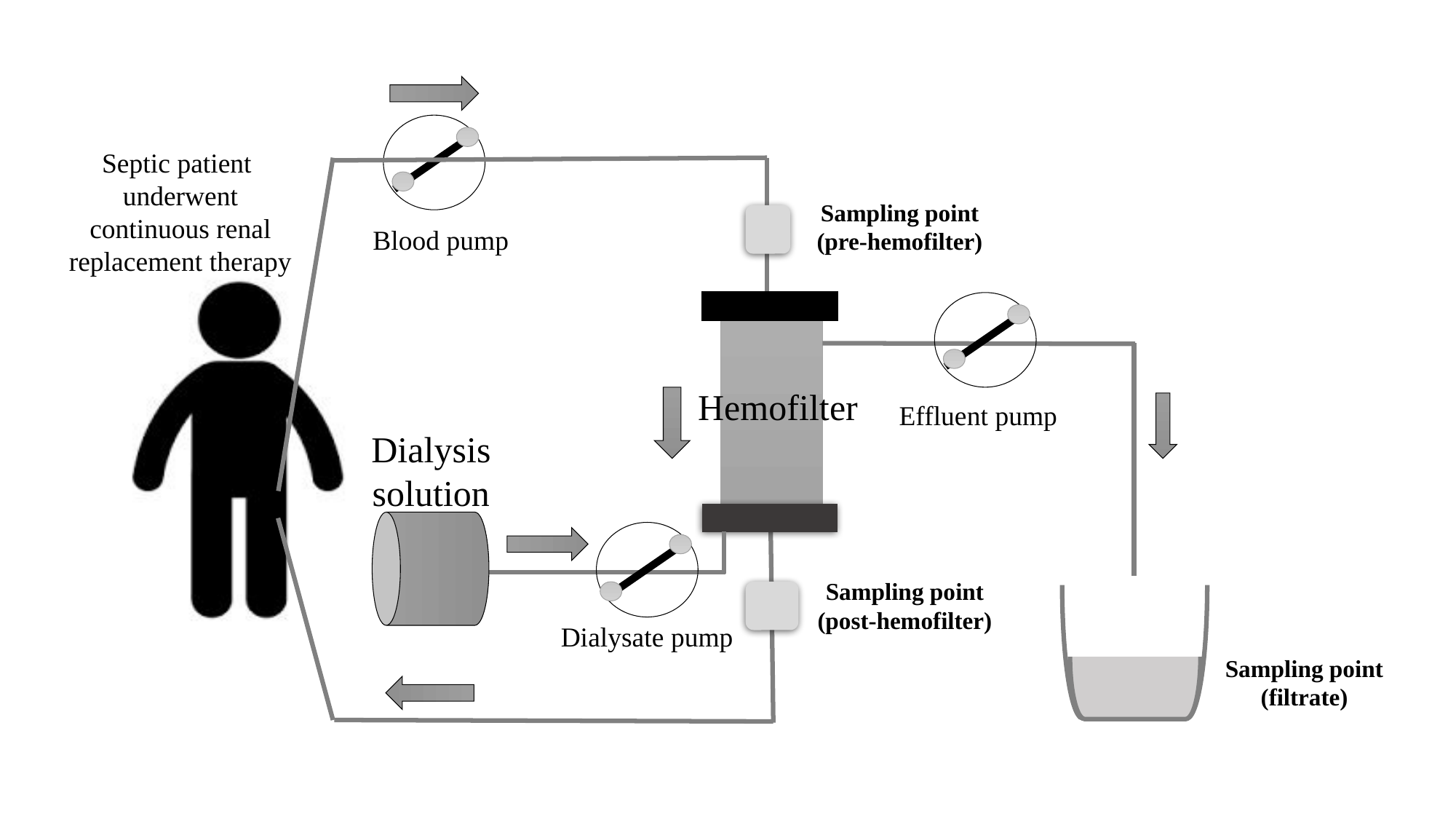

Sampling point
(pre-hemofilter)
Septic patient underwent continuous renal replacement therapy
Blood pump
Hemofilter
Effluent pump
Dialysis solution
Sampling point
(post-hemofilter)
Dialysate pump
Sampling point
(filtrate)
