# Supplementary material for: Comparison of the cytokine adsorption ability in continuous renal replacement therapy using polyethyleneimine-coated polyacrylonitrile (AN69ST) or polymethylmethacrylate (PMMA) hemofilters: a pilot single-center open-label randomized control trial
Source: Eur J Med Res. 2023 Jun 30;28:208. doi: 10.1186/s40001-023-01184-6 (PMC10314474; doi:10.1186/s40001-023-01184-6)
Supplement: Supplementary file 2 — Additional file 2. Cytokine levels at each sampling point. [file 40001_2023_1184_MOESM2_ESM.docx]

**Additional file 2.** Cytokine levels at each sampling point

|  |  |  | AN69ST | PMMA | *P* value*^a^* |
| --- | --- | --- | --- | --- | --- |
| Mediators | Sampling time window | Sampling point | (n = 26) | (n = 26) |  |
| HMGB1 (ng/mL) | 2–6 h | pre-hemofilter | 3.6 (2.0–5.5) | 3.1 (1.9–6.6) | 0.88 |
|  |  | post-hemofilter | 2.2 (1.2–4.0) | 4.3 (1.9–6.8) | < 0.05 |
|  |  | filtrate | 0 (0–0) | 0 (0–0.2) | 0.15 |
|  | 12–24 h | pre-hemofilter | 3.2 (2.3–5.5) | 3.9 (2.2–4.8) | 0.73 |
|  |  | post-hemofilter | 2.5 (1.4–3.7) | 4.1 (2.2–7.7) | < 0.05 |
|  |  | filtrate | 0 (0–0.4) | 0 (0–0.5) | 0.73 |
| TNF-α (pg/mL) | 2–6 h | pre-hemofilter | 2.9 (1.3–6.3) | 2.6 (1.3–5.6) | 0.69 |
|  |  | post-hemofilter | 2.1 (0.8–4.0) | 1.9 (0.9–4.9) | 0.76 |
|  |  | filtrate | 0.02 (0–0.03) | 0.01 (0–0.05) | 0.87 |
|  | 12–24 h | pre-hemofilter | 2.5 (1.7–4.7) | 2.2 (1.3–5.1) | 0.73 |
|  |  | post-hemofilter | 1.9 (1.2–3.7) | 1.8 (1.1–4.0) | 0.92 |
|  |  | filtrate | 0.03 (0–0.04) | 0 (0–0.01) | < 0.01 |
| IL-6 (pg/mL) | 2–6 h | pre-hemofilter | 1736.2 (584.0–16407.0) | 1369.4 (303.4–3791.8) | 0.48 |
|  |  | post-hemofilter | 1752.6 (504.2–15823.0) | 1209.3 (304.4–3373.8) | 0.38 |
|  |  | filtrate | 623.3 (206.7–6597.0) | 0.17 (0–1.2) | < 0.001 |
|  | 12–24 h | pre-hemofilter | 509.8 (182.0–7141.0) | 811.3 (172.1–2175.4) | 0.85 |
|  |  | post-hemofilter | 478.8 (189.3–6963.0) | 699.5 (145.4–2397.6) | 0.91 |
|  |  | filtrate | 116.5 (49.8–756.2) | 8.8 (1.1–30.2) | < 0.001 |
| IL-8 (pg/mL) | 2–6 h | pre-hemofilter | 96.9 (27.6–414.5) | 60.2 (34.3–324.3) | 0.69 |
|  |  | post-hemofilter | 53.6 (14.8–190.1) | 72.1 (34.5–315.6) | 0.21 |
|  |  | filtrate | 3.7 (1.7–6.6) | 30.8 (8.4–96.7) | < 0.001 |
|  | 12–24 h | pre-hemofilter | 57.2 (21.1–128.7) | 51.8 (33.1–81.8) | 0.74 |
|  |  | post-hemofilter | 52.4 (13.5–120.7) | 67.3 (34.9–112.9) | 0.2 |
|  |  | filtrate | 4.5 (2.6–11.7) | 60.7 (21.6–212.1) | < 0.001 |
| IL-10 (pg/mL) | 2–6 h | pre-hemofilter | 34.1 (14.0–142.1) | 47.1 (25.9–149.9) | 0.24 |
|  |  | post-hemofilter | 22.4-11.6–102.5) | 37.8 (21.2–97.4) | 0.14 |
|  |  | filtrate | 0.02 (0–0.13) | 0 (0–0.02) | < 0.01 |
|  | 12–24 h | pre-hemofilter | 23.8 (15.8–56.1) | 29.4 (17.5–84.9) | 0.41 |
|  |  | post-hemofilter | 21.6 (10.9–39.7) | 22.4 (15.2–73.7) | 0.26 |
|  |  | filtrate | 0.09 (0.03–0.28) | 0.06 (0–0.14) | < 0.05 |
| IL-18 (pg/mL) | 2–6 h | pre-hemofilter | 868.6 (553.1–1286.8) | 637.6 (516.5–1390.2) | 0.62 |
|  |  | post-hemofilter | 889.7 (595.1–1407.5) | 708.6 (587.2–1414.3) | 0.66 |
|  |  | filtrate | 1.3 (0.9–2.1) | 15.1 (7.2–28.8) | < 0.001 |
|  | 12–24 h | pre-hemofilter | 876.7 (681.6–2114.8) | 728.9 (602.4–1821.1) | 0.46 |
|  |  | post-hemofilter | 944.3 (715.6–2328.5) | 816.4 (660.3–2058.2) | 0.52 |
|  |  | filtrate | 0.93 (0.32–1.93) | 15.7 (5.3–28.6) | < 0.001 |
| MIG (pg/mL) | 2–6 h | pre-hemofilter | 76.8 (46.9–238.8) | 107.1 (41.6–438.6) | 0.52 |
|  |  | post-hemofilter | 18.1 (8.4–46.0) | 78.5 (30.5–357.3) | < 0.001 |
|  |  | filtrate | 1.7 (0.8–4.7) | 5.3 (1.1–27.1) | 0.06 |
|  | 12–24 h | pre-hemofilter | 51.5 (32.7–219.7) | 72.2 (33.5–334.6) | 0.52 |
|  |  | post-hemofilter | 14.1 (10.1–52.2) | 71.5 (28.1–313.6) | < 0.001 |
|  |  | filtrate | 3.8 (2.1–12.3) | 17.8 (8.8–51.2) | < 0.05 |
| MIP-1α (pg/mL) | 2–6 h | pre-hemofilter | 146.5 (72.2–323.8) | 149.6 (66.2–242.3) | 0.60 |
|  |  | post-hemofilter | 38.0 (16.4–74.1) | 84.8 (51.5–175.4) | < 0.05 |
|  |  | filtrate | 0.45 (0.17–1.85) | 0.14 (0.01–0.47) | < 0.01 |
|  | 12–24 h | pre-hemofilter | 108.1 67.2–180.7) | 90.9 (45.6–231.8) | 0.64 |
|  |  | post-hemofilter | 39.8 (20.3–74.5) | 74.8 (38.1–179.7) | < 0.05 |
|  |  | filtrate | 6.4 (0.9–10.4) | 2.2 (0.2–10.4) | 0.21 |

Data are given as medians and interquartile ranges.

*^a^* Wilcoxon test

AN69ST, polyethyleneimine-coated polyacrylonitrile; PMMA, polymethylmethacrylate; HMGB1, high-mobility group box 1; TNF, tumor necrosis factor; IL, interleukin; MIG, monokine induced by INFγ; MIP-1α, macrophage inflammatory protein 1 alpha
