# Supplementary material for: Comparison of the cytokine adsorption ability in continuous renal replacement therapy using polyethyleneimine-coated polyacrylonitrile (AN69ST) or polymethylmethacrylate (PMMA) hemofilters: a pilot single-center open-label randomized control trial
Source: Eur J Med Res. 2023 Jun 30;28:208. doi: 10.1186/s40001-023-01184-6 (PMC10314474; doi:10.1186/s40001-023-01184-6)
Supplement: Supplementary file 3 — Additional file 3. Number of serious adverse events. [file 40001_2023_1184_MOESM3_ESM.docx]

**Additional file 3.** Number of serious adverse events

|  | AN69ST | PMMA |
| --- | --- | --- |
|  | (n = 26) | (n = 26) |
| Serious adverse events, n |  |  |
| Shock | 0 | 0 |
| Disequilibrium syndrome | 0 | 0 |
| Anaphylaxis | 0 | 0 |
| Non-serious adverse events, n |  |  |
| Respiratory | 0 | 0 |
| Cardiovascular | 0 | 0 |
| Blood pressure reduction | 0 | 2 |
| Blood | 0 | 0 |
| Hyperkalemia | 0 | 1 |
| Thrombocytopenia | 1 | 0 |
| Nasal bleeding | 1 | 0 |
| Bleeding due to catheter insertion | 0 | 1 |
| Central nervous system | 0 | 0 |
| Others | 0 | 0 |

PMMA, polymethyl methacrylate; AN69ST, polyethyleneimine-coated polyacrylonitrile
