# Supplementary material for: Comparison of the cytokine adsorption ability in continuous renal replacement therapy using polyethyleneimine-coated polyacrylonitrile (AN69ST) or polymethylmethacrylate (PMMA) hemofilters: a pilot single-center open-label randomized control trial
Source: Eur J Med Res. 2023 Jun 30;28:208. doi: 10.1186/s40001-023-01184-6 (PMC10314474; doi:10.1186/s40001-023-01184-6)
Supplement: Supplementary file 4 — Additional file 4. Molecular weights and isoelectric points of cytokines. The theoretical molecular weights and isoelectric points of each cytokine are indicated in the Table and Figure***. Values were calculated by Expasy (https://web.expasy.org/compute_pi/) based on the amino acid sequence of matured protein. HMGB-1 high-mobility group box 1, TNF-α tumor necrosis factor, IL interleukin, MIG monokine induced by interferon-γ, MIP macrophage inflammatory protein. [file 40001_2023_1184_MOESM4_ESM.pptx]

## Slide 1
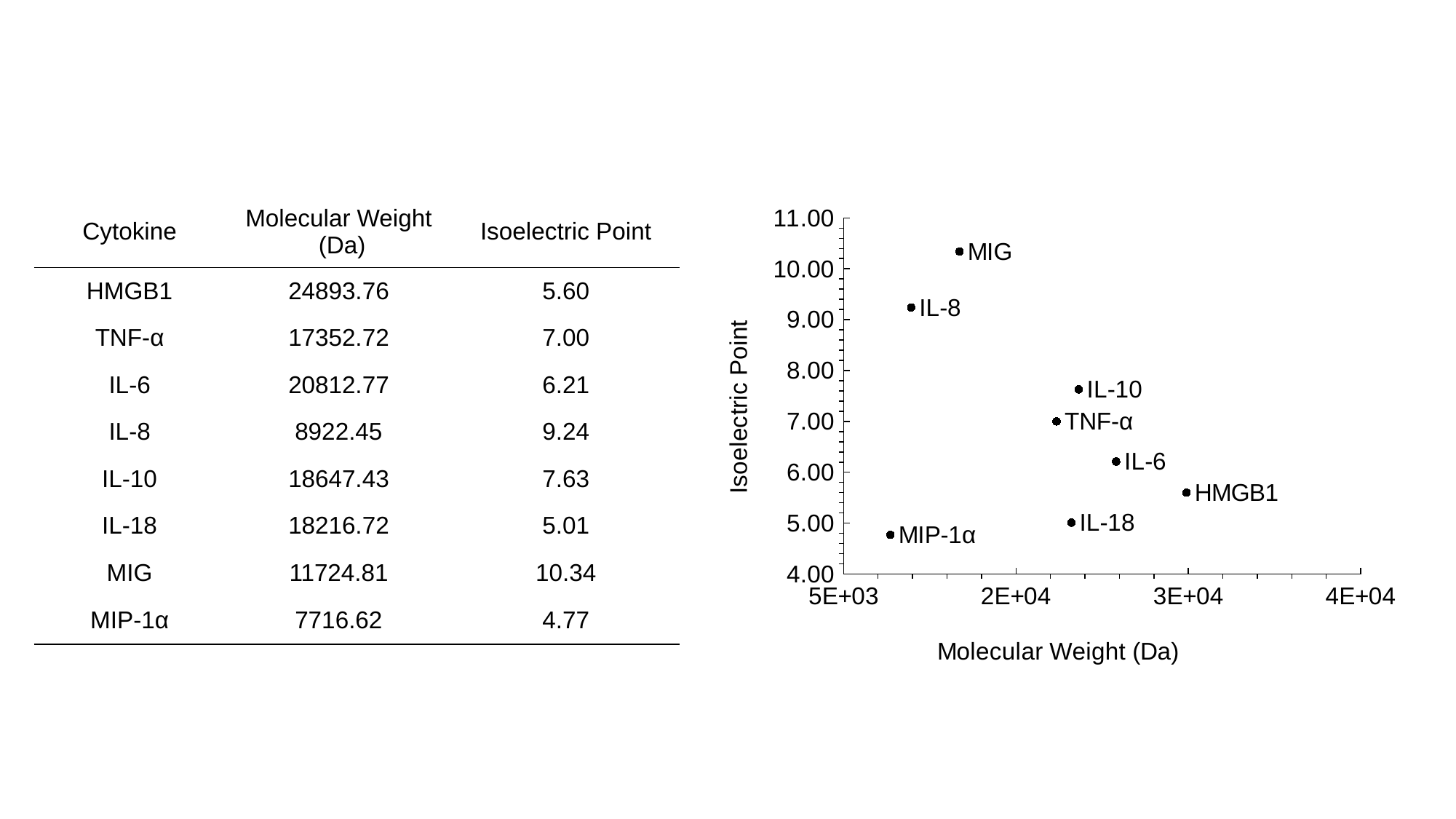

| Cytokine | Molecular Weight (Da) | Isoelectric Point |
| --- | --- | --- |
| HMGB1 | 24893.76 | 5.60 |
| TNF-α | 17352.72 | 7.00 |
| IL-6 | 20812.77 | 6.21 |
| IL-8 | 8922.45 | 9.24 |
| IL-10 | 18647.43 | 7.63 |
| IL-18 | 18216.72 | 5.01 |
| MIG | 11724.81 | 10.34 |
| MIP-1α | 7716.62 | 4.77 |
### Chart
| Category | |
|---|---|
