# Supplementary material for: Comparison of the cytokine adsorption ability in continuous renal replacement therapy using polyethyleneimine-coated polyacrylonitrile (AN69ST) or polymethylmethacrylate (PMMA) hemofilters: a pilot single-center open-label randomized control trial
Source: Eur J Med Res. 2023 Jun 30;28:208. doi: 10.1186/s40001-023-01184-6 (PMC10314474; doi:10.1186/s40001-023-01184-6)
Supplement: Supplementary file 5 — Additional file 5. Results of primary endpoint analysis after excluding patients with circuit life span of within 24 h. [file 40001_2023_1184_MOESM5_ESM.docx]

**Additional file 5.** Primary endpoint after excluding patients with circuit life span of up to 24 h (cytokine clearance of hemofilter adsorption)

|  |  |  | AN69ST | PMMA | *P* value*^a^* |
| --- | --- | --- | --- | --- | --- |
| Mediators | Clearance | Sampling time window | (n = 17) | (n = 18) |  |
| HMGB1 | Plasma clearance (mL/min) | 2–6 h | 32.7 (32.9–67.9) | -1.0 (-28.0–16.8) | < 0.001 |
|  |  | 12–24 h | 40.4 (9.9–49.1) | 0.9 (-60.6–14.0) | < 0.01 |
|  | Transmembrane clearance (mL/min) | 2–6 h | 0 (0–0) | 0 (0–0.4) | 0.16 |
|  |  | 12–24 h | 0 (0–0.34) | 0 (0–0.1) | 0.28 |
|  | Clearance of hemofilter adsorption (mL/min) | 2–6 h | 48.3 (32.6–67.9) | -1.5 (-28.3–16.5) | < 0.001 |
|  |  | 12–24 h | 40.4 (9.3–49.0) | -0.7 (-60.7–14.0) | < 0.01 |
| TNF-α | Plasma clearance (mL/min) | 2–6 h | 34.0 (30.2–38.4) | 27.4 (7.5–30.5) | < 0.01 |
|  |  | 12–24 h | 21.3 (17.4–25.6) | 12.0 (4.9–24.1) | < 0.05 |
|  | Transmembrane clearance (mL/min) | 2–6 h | 0.01 (0–0.05) | 0.02 (0–0.1) | 0.71 |
|  |  | 12–24 h | 0.04 (0–0.08) | 0.003 (0–0.06) | 0.24 |
|  | Clearance of hemofilter adsorption (mL/min) | 2–6 h | 33.9 (30.1–38.4) | 27.4 (7.4–30.5) | < 0.01 |
|  |  | 12–24 h | 21.3 (17.3–25.5) | 12.0 (4.8–24.1) | 0.07 |
| IL-6 | Plasma clearance (mL/min) | 2–6 h | 9.2 (8.1–11.1) | 16.7 (9.3–22.5) | < 0.01 |
|  |  | 12–24 h | 7.9 (3.6–9.7) | 8.2 (2.5–13.0) | 0.74 |
|  | Transmembrane clearance (mL/min) | 2–6 h | 1.9 (1.7–2.3) | 0.001 (0–0.001) | < 0.001 |
|  |  | 12–24 h | 1.5 (1.2–2.1) | 0.14 (0.01–0.24) | < 0.001 |
|  | Clearance of hemofilter adsorption (mL/min) | 2–6 h | 7.4 (4.3–9.3) | 16.7 (9.3–22.4) | < 0.001 |
|  |  | 12–24 h | 6.2 (2.3–9.1) | 8.0 (2.2–12.9) | 0.37 |
| IL-8 | Plasma clearance (mL/min) | 2–6 h | 48.3 (38.1–50.9) | 7.1 (-8.6–12.0) | < 0.001 |
|  |  | 12–24 h | 35.2 (20.6–48.4) | 3.4 (-16.7–10.5) | < 0.001 |
|  | Transmembrane clearance (mL/min) | 2–6 h | 0.10 (0.04–0.43) | 1.3 (1.0–3.5) | < 0.001 |
|  |  | 12–24 h | 0.4 (0.3–0.9) | 4.1 (3.4–8.6) | < 0.001 |
|  | Clearance of hemofilter adsorption (mL/min) | 2–6 h | 48.3 (38.1–50.7) | 5.8 (-11.2–10.4) | < 0.001 |
|  |  | 12–24 h | 34.8 (20.3–46.0) | 0.1 (-25.3–6.7) | < 0.001 |
| IL-10 | Plasma clearance (mL/min) | 2–6 h | 28.6 (25.2–35.8) | 26.7 (16.5–30.3) | 0.10 |
|  |  | 12–24 h | 23.7 (17.1–28.8) | 18.2 (12.8–23.2) | 0.10 |
|  | Transmembrane clearance (mL/min) | 2–6 h | 0 (0–0.01) | 0 (0–0) | 0.11 |
|  |  | 12–24 h | 0.02 (0.01–0.63) | 0.01 (0–0.01) | < 0.01 |
|  | Clearance of hemofilter adsorption (mL/min) | 2–6 h | 28.6 (25.2–35.8) | 26.7 (16.5–30.3) | 0.10 |
|  |  | 12–24 h | 23.7 (16.9–28.8) | 18.2 (12.8–23.2) | 0.10 |
| IL-18 | Plasma clearance (mL/min) | 2–6 h | -0.4(-4.7–2.2) | -1.9 (-3.0–0.2) | 0.32 |
|  |  | 12–24 h | 0.31 (-3.7–3.4) | -2.6 (-5.1–1.9) | 0.25 |
|  | Transmembrane clearance (mL/min) | 2–6 h | 0.01 (0.003–0.02) | 0.09 (0.06–0.11) | < 0.001 |
|  |  | 12–24 h | 0.004 (0.001–0.007) | 0.05 (0.03–0.07) | < 0.001 |
|  | Clearance of hemofilter adsorption (mL/min) | 2–6 h | -0.42 (-4.7–2.2) | -1.9 (-3.2–0.2) | 0.28 |
|  |  | 12–24 h | 0.4 (-3.1–3.8) | -2.7 (-5.1–1.9) | 0.13 |
| MIG | Plasma clearance (mL/min) | 2–6 h | 65.9 (62.0–67.9) | 22.9 (17.5–33.3) | < 0.001 |
|  |  | 12–24 h | 58.8 (52.4–61.3) | 9.1 (6.3–19.2) | < 0.001 |
|  | Transmembrane clearance (mL/min) | 2–6 h | 0.14 (0.06–0.20) | 0.12 (0.05–0.76) | 0.62 |
|  |  | 12–24 h | 0.6 (0.4–0.9) | 2.0 (1.2–2.3) | < 0.05 |
|  | Clearance of hemofilter adsorption (mL/min) | 2–6 h | 65.8 (61.8–67.7) | 22.7 (16.8–32.6) | < 0.001 |
|  |  | 12–24 h | 58.2 (50.1–60.6) | 6.6 (5.2–17.7) | < 0.001 |
| MIP-1α | Plasma clearance (mL/min) | 2–6 h | 64.8 (63.5–66.2) | 41.9 (18.6–46.1) | < 0.001 |
|  |  | 12–24 h | 51.6 (47.9–55.3) | 20.9 (4.0–31.1) | < 0.001 |
|  | Transmembrane clearance (mL/min) | 2–6 h | 0.02 (0.01–0.04) | 0.01 (0.001–0.02) | < 0.05 |
|  |  | 12–24 h | 0.3 (0.15–0.69) | 0.25 (0.01–1.09) | 0.69 |
|  | Clearance of hemofilter adsorption (mL/min) | 2–6 h | 64.8 (63.5–66.2) | 40.2 (18.6–46.1) | < 0.001 |
|  |  | 12–24 h | 51.4 (47.1–55.2) | 20.6 (2.7–31.1) | < 0.001 |

Data are given as medians and interquartile ranges.

*^a^* Wilcoxon test

Plasma clearance = (Cbi - Cbo) / Cbi × (Qb - QF) + QF

Transmembrane clearance = CF / Cbi × QF

Clearance of hemofilter adsorption = Plasma clearance - Transmembrane clearance

Cbi, blood cytokine level at the filter inlet; Cbo, blood cytokine level at the outlet; Qb, blood flow rate (mL/min); QF, flow rate of the ultrafiltrate; CF, cytokine level in the filtrate

AN69ST, polyethyleneimine-coated polyacrylonitrile; PMMA, polymethylmethacrylate; HMGB1, high-mobility group box 1; TNF, tumor necrosis factor; IL, interleukin; MIG, monokine induced by interferon-γ; MIP-1α, macrophage inflammatory protein 1 alpha
